# Supplementary figures and images for: Impact of the period of the day on all-cause mortality and major cardiovascular complications after arterial vascular surgeries
Source: PLoS One. 2023 Jan 5;18(1):e0279873. doi: 10.1371/journal.pone.0279873 (PMC9815593; doi:10.1371/journal.pone.0279873)

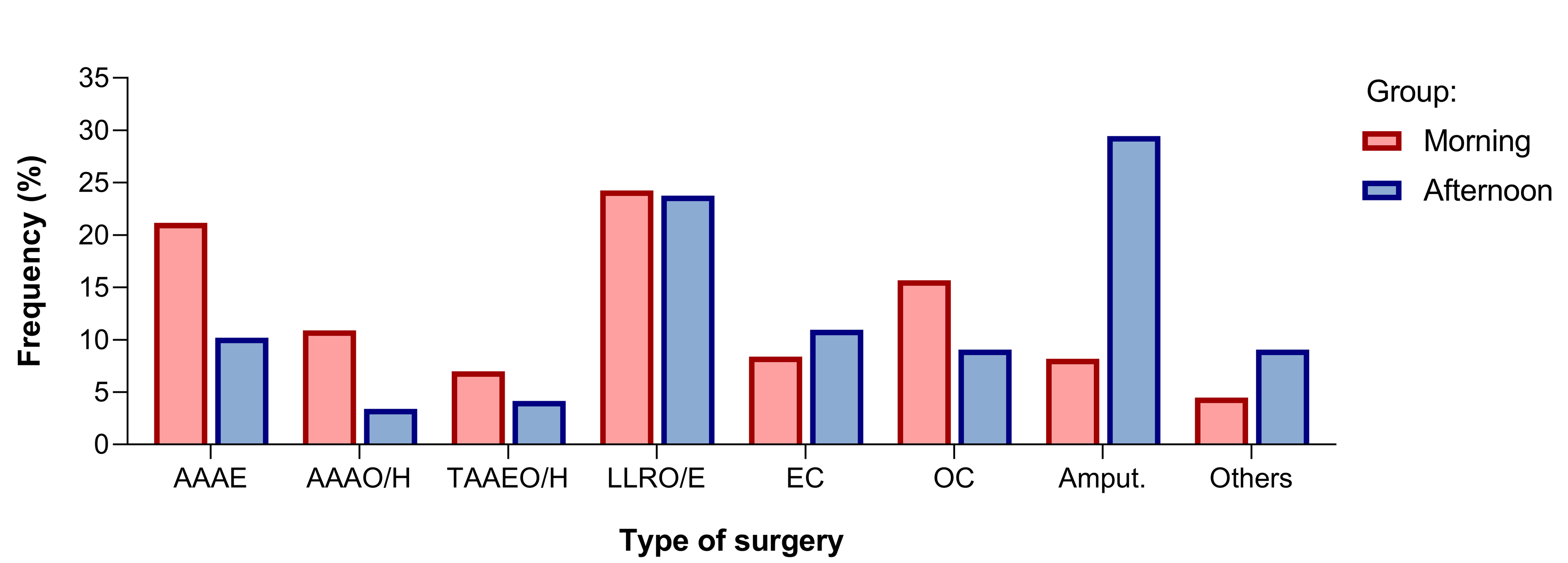

Supplement: S1 Fig — AAAE = Abdominal Aortic Aneurysm Endovascular; AAAO/H = Open or Hybrid Abdominal Aortic Aneurysm; TAAEO/H = Thoracic Aortic Aneurysm Endovascular, Open or Hybrid; LLRO/E = Open or Endovascular Lower Limb Revascularization; EC = Endovascular Carotid; OC = Open Carotid; AMPUT = Amputations; OTHERS = Open or Endovascular Visceral Arteries and Other Procedures. (TIF) [file pone.0279873.s001.tif]

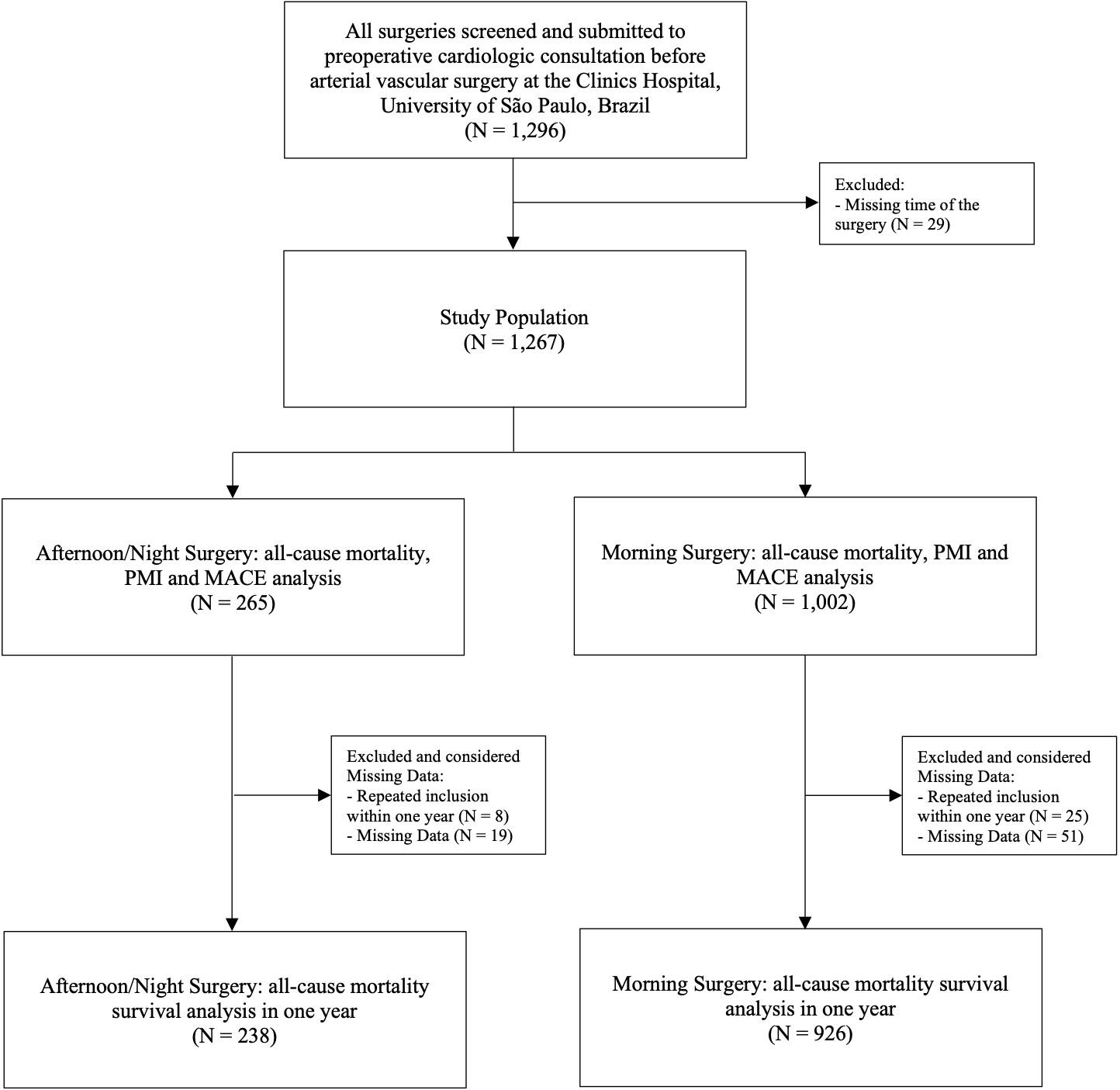

Supplement: S2 Fig — (TIF) [file pone.0279873.s002.tif]

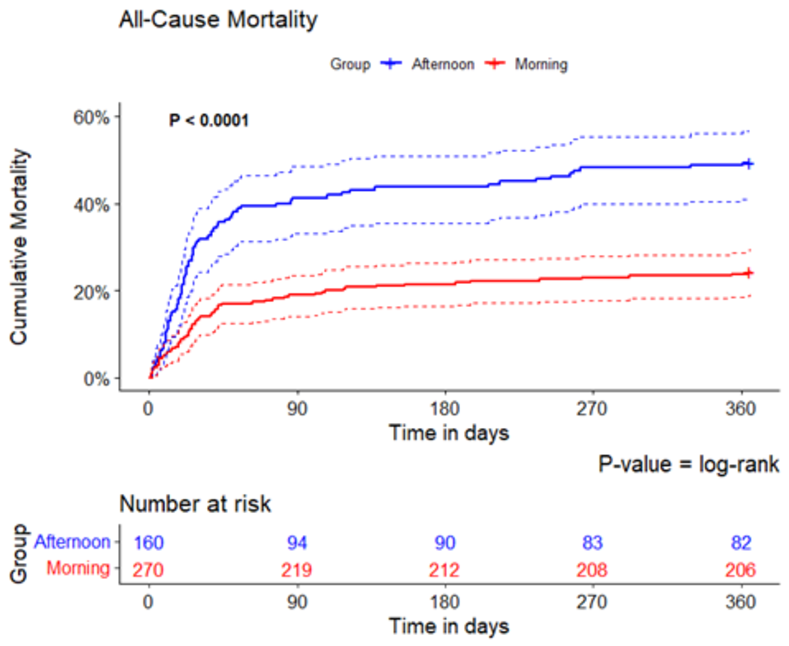

Supplement: S3 Fig — (TIF) [file pone.0279873.s003.tif]
