# Supplementary material for: Impact of the period of the day on all-cause mortality and major cardiovascular complications after arterial vascular surgeries
Source: PLoS One. 2023 Jan 5;18(1):e0279873. doi: 10.1371/journal.pone.0279873 (PMC9815593; doi:10.1371/journal.pone.0279873)
Supplement: S1 Table — (DOCX) [file pone.0279873.s004.docx]

**Supporting Table 1.** STROBE Statement—Checklist of items that should be included in reports of cohort studies

|  | **Item No** | **Recommendation** | **Page** |
| --- | --- | --- | --- |
| **Title and Abstract** | 1 | (*a*) Indicate the study’s design with a commonly used term in the title or the abstract. | 2 |
|  |  | (*b*) Provide in the abstract an informative and balanced summary of what was done and what was found. | 2 |
| **Introduction** | | |  |
| Background/rationale | 2 | Explain the scientific background and rationale for the investigation being reported. | 3 |
| Objectives | 3 | State specific objectives, including any prespecified hypotheses. | 3 |
| **Methods** | | |  |
| Study design | 4 | Present key elements of study design early in the paper. | 4 |
| Setting | 5 | Describe the setting, locations, and relevant dates, including periods of recruitment, exposure, follow-up, and data collection. | 4 |
| Participants | 6 | (*a*) Give the eligibility criteria, and the sources and methods of selection of participants. Describe methods of follow-up. | 4 |
|  |  | (*b*)For matched studies, give matching criteria and number of exposed and unexposed. | - |
| Variables | 7 | Clearly define all outcomes, exposures, predictors, potential confounders, and effect modifiers. Give diagnostic criteria, if applicable. | 5 |
| Data sources/measurement | 8* | For each variable of interest, give sources of data and details of methods of assessment (measurement). Describe comparability of assessment methods if there is more than one group. | 6 |
| Bias | 9 | Describe any efforts to address potential sources of bias. | 6 |
| Study size | 10 | Explain how the study size was arrived at. | - |
| Quantitative variables | 11 | Explain how quantitative variables were handled in the analyses. If applicable, describe which groupings were chosen and why. | 6 |
| Statistical methods | 12 | (*a*) Describe all statistical methods, including those used to control for confounding. | 6 |
|  |  | (*b*) Describe any methods used to examine subgroups and interactions. | - |
|  |  | (*c*) Explain how missing data were addressed. | 6 |
|  |  | (*d*) If applicable, explain how loss to follow-up was addressed. | 6 |
|  |  | (*e*) Describe any sensitivity analyses. | - |
| **Results** | | |  |
| Participants | 13* | (a) Report numbers of individuals at each stage of study—e.g. numbers potentially eligible, examined for eligibility, confirmed eligible, included in the study, completing follow-up, and analyzed. | 7 |
|  |  | (b) Give reasons for non-participation at each stage. | 7 |
|  |  | (c) Consider use of a flow diagram. | 7 |
| Descriptive data | 14* | (a) Give characteristics of study participants (e.g. demographic, clinical, social) and information on exposures and potential confounders. | 7-8 |
|  |  | (b) Indicate number of participants with missing data for each variable of interest. | 8 |
|  |  | (c) Summarise follow-up time (eg, average and total amount). | 8 |
| Outcome data | 15* | Report numbers of outcome events or summary measures over time. | 9 |
| Main results | 16 | (*a*) Give unadjusted estimates and, if applicable, confounder-adjusted estimates and their precision (eg, 95% confidence interval). Make clear which confounders were adjusted for and why they were included. | 10 |
|  |  | (*b*) Report category boundaries when continuous variables were categorized. | - |
|  |  | (*c*) If relevant, consider translating estimates of relative risk into absolute risk for a meaningful time period. | - |
| Other analyses | 17 | Report other analyses done—e.g. analyses of subgroups and interactions, and sensitivity analyses. | - |
| **Discussion** | | |  |
| Key results | 18 | Summarise key results with reference to study objectives. | 12 |
| Limitations | 19 | Discuss limitations of the study, taking into account sources of potential bias or imprecision. Discuss both direction and magnitude o fany potential bias. | 14 |
| Interpretation | 20 | Give a cautious overall interpretation of results considering objectives, limitations, multiplicity of analyses, results from similar studies, and other relevant evidence. | 13 |
| Generalizability | 21 | Discuss the generalizability (external validity) of the study results. | 13-14 |
| **Other information** | | |  |
| Funding | 22 | Give the source of funding and the role of the funders for the present study and, if applicable, for the original study on which the present article is based. | 16 |
