# Supplementary material for: Impact of the period of the day on all-cause mortality and major cardiovascular complications after arterial vascular surgeries
Source: PLoS One. 2023 Jan 5;18(1):e0279873. doi: 10.1371/journal.pone.0279873 (PMC9815593; doi:10.1371/journal.pone.0279873)
Supplement: Attachment — Submitted filename: Response to Reviewers.docx [file pone.0279873.s005.docx]

**Rebuttal Letter**

Impact of the period of the day on all-cause mortality and major cardiovascular complications after arterial vascular surgeries

Dear Editor and Reviewers,

We appreciate your patience and consideration on reviewing our manuscript. It is an honor for us to have the opportunity to publish in PLOS ONE. We have carefully read your comments and we changed the manuscript accordingly.

Please, note that changes we made in the manuscript text are marked as the “red” color. Also, we are providing the files of both marked and clean “Impact of the period of the day on all-cause mortality and major cardiovascular complications after arterial vascular surgeries” reviewed versions attached.

**List of Changes – Journal Requirements**

**1. Editor:** Please ensure that your manuscript meets PLOS ONE's style requirements, including those for file naming.

**Authors’ Feedback:** As recommended by the Editor, we have revised the requirements in our manuscript regarding style, file naming and references, and we believe it is now correct.

**2. Editor:** Please provide additional details regarding participant consent. In the Methods section, please ensure that you have specified (1) whether consent was informed and (2) what type you obtained (for instance, written or verbal). If your study included minors, state whether you obtained consent from parents or guardians. If the need for consent was waived by the ethics committee, please include this information.

**Authors’ Feedback:** The ethics committee waived the need for an informed consent in our study, because it involved analysis of medical records from the Hospital database. We have added the following sentence to the study.

Methods (page 5, line 124): “The need for Informed Consent was waived by the ethics committee.”

**3. Editor:** Please confirm that this does not alter your adherence to all PLOS ONE policies on sharing data and materials, by including the following statement: "This does not alter our adherence to PLOS ONE policies on sharing data and materials.” (as detailed online in our guide for authors <http://journals.plos.org/plosone/s/competing-interests>).  If there are restrictions on sharing of data and/or materials, please state these. Please note that we cannot proceed with consideration of your article until this information has been declared. Please include your updated Competing Interests statement in your cover letter; we will change the online submission form on your behalf.

**Authors’ Feedback:** As recommended by the Editor, we have added the sentence "this does not alter our adherence to PLOS ONE policies on sharing data and materials” in the Competing Interests section in our cover letter.

**4. Editor:** Please include captions for your Supporting Information files at the end of your manuscript, and update any in-text citations to match accordingly. Please see our Supporting Information guidelines for more information: http://journals.plos.org/plosone/s/supporting-information.

**Authors’ Feedback:** As recommended by the Editor, we have revised the requirements in our manuscript regarding captions for Supporting Information and citations, and we believe it is now correct.

**List of Changes – Reviewers’ Comments**

**Reviewer #1**

1. **Reviewer:** Distribution with surgery schedule? An histogram of the distribution of surgery time-of-the-day should be provided.

**Authors’ Feedback:** Based on the surgical work shifts at our Hospital, predefined for the present study, we have categorized the variable time of the surgery into two groups: morning surgeries (between 7 a.m. to 12 a.m.) and afternoon/night surgeries (between 12:01p.m. to 6:59a.m. of the next day). We believe that this categorical approach might be more robust than a continuous one. Also, we believe that the categorical approach has more clinical relevance due to its feasibility and capacity to be more understandable, and it’s more related to Hospital work shifts. The histogram was added in the Suporting Material of the Manuscript.

2. **Reviewer:** Divided Afternoon & night surgery.

**Authors’ Feedback:** The decision to piece together afternoon and night surgeries was made based on our Hospital work shifts of the surgical teams. Also, night surgeries mostly included urgent/emergency procedures and it didn’t represent a significative number of surgeries so we could split and without jeopardizing the power of the study. Therefore, we made the choice to categorize it in “afternoon/night” surgeries (12:01p.m. to 6:59a.m. of the next day).

3. **Reviewer:** Recently Montaigne et al. published a paper regarding time-of-the-day and Kidney transplantation which should be discussed in line with the conclusion. It would be of interest to compare day-time and night surgery, by different medical organization?

**Authors’ Feedback:** Many thanks to the reviewer for the suggestion. Despite Montaigne’s article is related to a different surgical procedure, the idea of comparing surgical outcomes between two different period of the day is in alignment with the present study. However, as we stated in item 2, the periods of the day that were compared are different, in our study and in Montaigne’s study. As stated in item 2, in our study the choice of the period of the day to be compared were related to Hospital work shifts of the surgical team.

4. **Reviewer:** Line 78: The sentence is quite unclear. “it is poorly understood if daytime variation also occurs in the perioperative period”, please rephrase.

**Authors’ Feedback:** We agree with the suggestion made by the reviewer. We have corrected the sentence in order to make it clearer and better understandable. The modified version is shown below:

Introduction (page 3, line 74): “However, there is still a gap in the literature assessing daytime variation of cardiovascular outcomes in the perioperative period.”

5. **Reviewer:** Line 130 to 134: In the paper of du Fay and colleagues and Montaigne and colleagues, an interval of 3 hours was observed between “morning” and “afternoon”.

**Authors’ Feedback:** We agree with the comments. To avoid confusion, we have corrected the sentence in page 5, line 128. Additionally, we decided to maintain our definition of morning surgeries (between 7 a.m. to 12 a.m.) and afternoon/night surgeries (between 12:01p.m. to 6:59a.m. of the next day) because we wanted to gather the maximum number of surgeries as possible. Considering the distribution of the time of our surgeries, very few happened in the splitting time (for example around 6a.m. or 12a.m.), and so this pattern wouldn’t underpower our categorization or results.

Methods (page 5, line 131): “These time intervals reflect the Hospital work shifts of the surgical team at our study center and are an example of the pattern of categorization between morning and afternoon surgeries previously described in the literature [15, 16].”

6. **Reviewer:** Line 154: What is “considered clinically significant”? High incidence of atrial fibrillation in the morning could distort the observations.

**Authors’ Feedback:** Clinically significant arrhythmias were defined as those in need of active drug therapy or electrical cardioversion. This definition was stablished to assess arrhythmias (one of the topics of Major Adverse Cardiac Events – MACE) which were adverse events after the arterial vascular surgeries. To make it clearer, we have added the following.

Methods (page 5, line 146): “…new or decompensated arrhythmias requiring treatment…”

7. **Reviewer:** Line 202: What type of vascular surgery? Duration of the operation?

**Authors’ Feedback:** We included patients undergoing all arterial vascular surgeries: open or endovascular (for aorta, peripheral artery, visceral artery, and carotid artery diseases and amputations due to limb ischemia), emergent, urgent, or elective. The distribution by surgery type is similar when comparing morning and afternoon, not considering urgent/emergency surgeries, which were analyzed as a confounding variable in our analysis. We have added the histogram in the Supporting Material of the Manuscript.

Results (page 8, line 220): “…arterial vascular surgery…”

8. **Reviewer:** Table 1: Please provide preoprative TnT and Left ventricular ejection fraction?

**Authors’ Feedback:** In our Hospital, preoperative Echocardiogram and cardiac troponin levels are not part of the routine exams. These exams are performed at physician’s discretion. We have checked and the minority of the patients had these exams available.

9. **Reviewer:** No exclusion criterias, why? No flowchart?

**Authors’ Feedback:** We determined that the exclusion criteria of our study is, as stated in the methodology and in the Supportive Figure 1 (our flowchart): “For this analysis, we excluded patients from whom it was not possible to obtain time of the surgery with the available documentation.” (Methods page 4, line 101) We considered that other situations, as mentioned by the reviewer in the suggestions, are contraindications for surgical procedures, not exclusion criteria.

10. **Reviewer:** Emergency surgery represent a high proportion of the afternoon group vs morning group and weights a lot in the Cox regression. Why is this particular population not excluded? This is a major confounding factor that should be presented as a clear limitation.

**Authors’ Feedback:** We thank the reviewer for the comment. However, our group decided to include urgent/emergency surgeries in our cohort because of some factors. First, urgent/emergency surgeries represent an important share of arterial vascular surgeries in our Hospital. In this way, we wanted to study the same endpoints than in elective surgeries to have a perception of how they would perform equally or differently. Second, although the proportion of urgent/emergency surgeries could weight in the Cox Regression, we considered that our Regression Model and population was strong enough to estimate the effect size of each variable/confounder, allowing to identify the interaction effects between treatment and confounders. Lastly, as recommended by the reviewer, we have added this discussion in the manuscript text.

Discussion (page 13, line 292): “Also, urgent/emergency procedures were more prevalent than in the other studies. We decided to include urgent/emergency surgeries in our cohort because they represent an important share of arterial vascular surgeries in our service. In this way, we wanted to study the same endpoints than in elective surgeries to have a perception of how they would perform. Although the proportion of urgent/emergency surgeries could weight in the Cox Regression, we considered that our Regression Model and population was strong enough to estimate the effect size of each variable/confounder.”

11. **Reviewer:** Fasting duration could be important in peri-operative studies, because of the amount of ketone bodies increasing in time. What was the fasting durations in each group (including emergency surgery)? Was the ketonemia higher in any group? This should be at least discussed. (Youm et al. Nat. Med. 2015)

**Authors’ Feedback:** It is true that fasting duration is an important factor for perioperative care. However, it is not routine in our Hospital service to previously measure ketone bodies in surgical patients. For elective surgeries, we have the protocol of 8 hours of fasting duration pre surgery. We don’t have the fasting durations in urgent/emergency surgeries. We added this to the Discussion and Limitation as described below.

Discussion (page 14, line 311): “Finally, for elective surgeries, our hospital have the protocol of 8 hours of fasting before the procedure. However, due to delays and other non-measured factors patients might have been exposed to longer periods of fasting, especially for afternoon/night procedures. Prolonged fasting might have contributed to the observed outcomes [26, 27]. The fasting period of urgent/emergency surgeries was not measured.”

12. **Reviewer:** Is nosocomial infection an exclusion criteria from this cohort?

**Authors’ Feedback:** The presence of nosocomial infection was not an exclusion criteria for our study.

13. **Reviewer:** In this population (>1200 patients) case-control with propensity matching design would be of interest.

**Authors’ Feedback:** We have considered Propensity Score Matching as a possibility to our statistical analysis as it could be an easier method to discuss with the audience, more straightforward and objective. It was done in one of the papers in our references (du Fay and colleagues). However, we considered that Propensity Score Matching wouldn’t allow us to see clear interactions between treatment and confounders, and it wouldn’t allow us to use all patients in the cohort, losing some interactions we wanted to search and losing power. Also, Propensity Score Matching would be more feasible if the number of outcome events/number of confounders were less than 7, which is not the case of our study. Therefore, we believe that Cox Regression Model is suitable to our study. We have added those points in the methods section.

Methods (page 6, line 177): “Our Regression Model and population was considered strong enough to estimate the effect size of each variable/confounder, allowing to identify the interaction effects between treatment and confounders.”

**Reviewer #2**

1. **Reviewer:** This is an interesting paper describing increased postoperative mortality both at hospital discharge and one year following vascular surgery in a tertiary centre for operations started after 12 mid-day compared to those whose operation was started in the morning between 7am and mid-day. The paper illustrates well that those operated later in the day were sicker, higher cardiac risk and not surprisingly did less well immediately after surgery and even a year after (20.8% and 34.7% mortality !). It is disappointing therefore that in the design of the study (and these results might well have been expected -what was the hypothesis?) data was not included at base line which might address the reason why operations started later in the day have sicker patients, more often are urgent/ emergent and do less well. Some factors are raised in the discussion but this study does not address the system issues.

**Authors’ Feedback:** We thank the reviewer for the suggestion. We have categorized the variable time of the surgery into two groups based on the surgical work shifts at our Hospital: morning surgeries (between 7 a.m. to 12 a.m.) and afternoon/night surgeries (between 12:01 p.m. to 6:59 a.m. of the next day). The null hypothesis of our study is that there is no difference in outcomes between these two groups. We modified the final version of the manuscript to make it clearer.

2. **Reviewer:** The method describes that only Consecutive cases for whom a preoperative cardiological consultation was requested were included. There were 1267 patients but how many patients were there who didn’t have a cardiological consultation? Obtaining this consultation may be affected by time of day or urgency? Can the authors comment on this and preferably indicate how many other vascular surgical procedures were done without this consultation and what was the mortality rate for this group- to give a full picture of the context of this study.

**Authors’ Feedback:** We added this information as a Limitation in the final version of the manuscript.

Discussion (page 14, line 328): “We included in the cohort patients for whom cardiac consultation was requested, and although it is a common practice at our institution for vascular surgeons to request cardiac evaluation for all patients, we may have missed patients in the lower risk strata.”

3. **Reviewer:** In vascular surgical practice repeat operations are not uncommon how was this taken into account in the study.

**Authors’ Feedback:** We added the following sentence in the methods section, so it is clearer and better understandable.

Methods (page 4, line 110): “The patient was included in the register just once in the index operations. Reoperations in the same hospitalization were not included.

4. **Reviewer:** No distinction is made regarding the nature of the vascular procedures done – it would be important to describe these as the outcomes, the open vs endovascular options, major vs minor and choice of time of day will be influence by these.

**Authors’ Feedback:** Our group decided to categorize arterial vascular surgeries by its start time, apart from the type of the surgery. Based on the surgical service hours of our study center, we have categorized the variable time of the surgery into two groups: morning surgeries (between 7 a.m. to 12 a.m.) and afternoon/night surgeries (between 12:01p.m. to 6:59a.m. of the next day). We believe that this categorical approach, considering all arterial vascular surgeries together, might be more robust, due to its clinical relevance, feasibility and capacity to be more understandable. Although this strategy could decrease the power of our study, our statistical methods were calculated enough to diminish this situation. We have added an histogram with surgeries type in the Supporting Material of the Manuscript.

5. **Reviewer:** Follow up was described both as 30 days and at -hospital discharge -which was it? One year data was by telephone and registries. In the Survival data there are multiple steps – does this mean telephone calls were made regularly?

**Authors’ Feedback:** All-cause mortality (primary endpoint) was followed-up during 30-days after the surgery and one year after the surgery. 30-days follow-up was made by in-hospital stay registries, postoperative consultation data or telephone calls. One year follow-up for all-cause mortality was made by telephone calls, patient’s charts and local death registries (with the appropriate death date confirmed), which explain why it was possible to obtain multiple steps in the Survival analysis.

PMI and MACE (secondary outcomes) were followed-up until hospital discharge by in-hospital stay registries.

We have corrected the text in the manuscript, so it is clearer and better understandable.

Methods (page 5, line 136): “30-days follow-up was made by in-hospital stay registries, postoperative consultation data or telephone calls. One year follow-up for all-cause mortality was made by telephone calls, patient’s charts and local death registries (with the appropriate death date confirmed).”

Methods (page 5, line 141): “PMI and MACE were followed-up until hospital discharge by in-hospital stay registries.”

6. **Reviewer:** The authors describe urgent/emergency surgery (how was this defined? and how many of these underwent prior cardiological assessment? This was a very major difference in the two time groups 68% compared to 29.6%. The data suggests that this was the most important risk factor maybe even more so than time of day.

**Authors’ Feedback:** Urgent/emergency procedures were considered surgeries non-previously scheduled, which means they were not elective and not previously followed up in ambulatorial treatment. Our group decided to include urgent/emergency surgeries in our cohort because of some factors. First, urgent/emergency surgeries represent an important share of arterial vascular surgeries in our service. In this way, we wanted to study the same endpoints than in elective surgeries to have a perception of how they would perform equally or differently. Second, although the proportion of urgent/emergency surgeries could weight in the Cox Regression, we considered that our Regression Model and population was strong enough to estimate the effect size of each variable/confounder, allowing to identify the interaction effects between treatment and confounders. Urgent/emergency surgery are an important risk factor, which was something previously expected. Lastly, as recommended by the reviewer, we have added this discussion in the manuscript text.

Methods (page 4, line 99): “Urgent/emergency procedures were considered surgeries non-previously scheduled, which means they were not elective and not recently evaluated in an outcome section.”

Discussion (page 13, line 292): “Also, urgent/emergency procedures were more prevalent than in the other studies. We decided to include urgent/emergency surgeries in our cohort because they represent a significant share of all vascular surgeries performed in our service. Therefore, we aimed to evaluate the same endpoints of elective surgeries to have a perception of how they would perform. Although the proportion of urgent/emergency surgeries could weight in the Cox Regression, we considered that our Regression Model and population was strong enough to estimate the effect size of each variable/confounder.”

7. **Reviewer:** Most of the analysis is of patient characteristics and they were sicker but what of other system factors that would affect the time distribution? Was there any access to operating theatre issue – which pushed procedures in to the afternoon. Were there delays to surgery which pushed procedures to later in the day. Interestingly urgency was a major risk factor for MACE.

**Authors’ Feedback:** We decided to focus on patients’ characteristics and clinical features to make our results more significant to clinical practice. Unfortunately, we didn’t have access to reasons for delays and operating issues. Additionally, we agree that this should be discussed as a limitation. We added the following in our manuscript text.

Discussion (page 14, line 303): “Not only patients’ characteristics and clinical aspects are responsible for time distribution; operating issues and surgeries delay could affect the schedule of the surgeries, which were not assessed in our study.”

8. **Reviewer:** The pattern of referral to this tertiary hospital was mentioned - as a possible system factor. Regional referral patterns for vascular surgery are well recognised, which may be related to availability of vascular surgery specialists and appropriate support services. If not in the data collected can the authors please comment on the factors in their hospital/region that influence what they have reported which could lead to outcome improvement.

**Authors’ Feedback:** We thank the reviewer for the comment. Our Institution is a tertiary University Hospital that deals with more complex surgical interventions like arterial vascular surgeries. In the Brazilian public health system, more complex interventions like those described in this study can occur only in specialized Hospitals. 100% of the patients in the present study are included in the Brazilian public health system.

9. **Reviewer:** Discussion page 12 line 271 it says “Additionally, our population were exclusive of patients undergoing arterial vascular surgery, which are patients at greater cardiac risk undergoing mostly high-risk operations. -could the authors please explain ? Do they mean Additionally, our population included only patients... Or Additionally, our population were exclusively patients undergoing…

**Authors’ Feedback:** We have corrected the sentence, so it is better represented.

Discussion (page 13, line 284): “We did not evaluate the incidence of MACE at long term follow-up, thus we could have missed some events. Also, since we had a higher-risk population with patients undergoing vascular surgeries that need cardiac referral, the elevated all-cause mortality rates observed may have overshadow other cardiac events.”

10. **Reviewer:** The Graphical abstract fig 2 should be revisited as it misses the point of the study – This reviewer would suggest that the study is not primarily about “need for better postoperative outcomes control” (not sure what that means). The message is about operations done later in the day – How can we insure the high risk patients are better prepared and more often operated earlier in the day.

**Authors’ Feedback:** We agree with the comment made by the reviewer. Therefore, we have updated our Fig 2 rephrasing “need for better postoperative outcomes control” to “Influence of surgery starting time on mortality and cardiac events”.

11. **Reviewer:** Similarly the Conclusion should be revisited – Vascular surgery patients operated later in the day are sicker, more urgent and have worse outcomes with double the mortality at 30m days and even out to one year. Steps are needed to improve outcomes which may include starting operations earlier.

**Authors’ Feedback:** We thank the reviewer for the comment but, in our opinion, the conclusion suits and responds our study objectives. The study design and the statistical analysis showed that vascular surgeries performed in the afternoon/night were independently associated with higher MACE and mortality in 30 days and one year. As suggested by the reviewer, we stated at the end of discussion section that prioritizing high-risk patients for operating during the morning may be a strategy for reducing cardiovascular complications.
