# Supplementary material for: Impact of the period of the day on all-cause mortality and major cardiovascular complications after arterial vascular surgeries
Source: PLoS One. 2023 Jan 5;18(1):e0279873. doi: 10.1371/journal.pone.0279873 (PMC9815593; doi:10.1371/journal.pone.0279873)
Supplement: Attachment — Submitted filename: Response to Reviewers 07-27-22.docx [file pone.0279873.s006.docx]

**Response to Reviewers**

**Impact of the period of the day on all-cause mortality and major cardiovascular complications after arterial vascular surgeries**

Dear Editor and Reviewers,

The first round of Review was really enriching for us. We appreciate your consideration on reviewing our manuscript. We have carefully read and analyzed your comments and provided answers and changes when necessary.

For this second round of Review, please note that changes we made in the manuscript text are marked as the “red” color. Also, we are providing the files of both marked and clean “Impact of the period of the day on all-cause mortality and major cardiovascular complications after arterial vascular surgeries” reviewed versions attached.

1. If the authors have adequately addressed your comments raised in a previous round of review and you feel that this manuscript is now acceptable for publication, you may indicate that here to bypass the “Comments to the Author” section, enter your conflict of interest statement in the “Confidential to Editor” section, and submit your "Accept" recommendation.

**Reviewer #1: All comments have been addressed**

**Reviewer #2: (No Response)**

2. Is the manuscript technically sound, and do the data support the conclusions?
The manuscript must describe a technically sound piece of scientific research with data that supports the conclusions. Experiments must have been conducted rigorously, with appropriate controls, replication, and sample sizes. The conclusions must be drawn appropriately based on the data presented.

**Reviewer #1: Yes**

**Reviewer #2: Partly**

3. Has the statistical analysis been performed appropriately and rigorously?

**Reviewer #1: Yes**

**Reviewer #2: Yes**

4. Have the authors made all data underlying the findings in their manuscript fully available?
The [PLOS Data policy](http://www.plosone.org/static/policies.action#sharing) requires authors to make all data underlying the findings described in their manuscript fully available without restriction, with rare exception (please refer to the Data Availability Statement in the manuscript PDF file). The data should be provided as part of the manuscript or its supporting information, or deposited to a public repository. For example, in addition to summary statistics, the data points behind means, medians and variance measures should be available. If there are restrictions on publicly sharing data—e.g. participant privacy or use of data from a third party—those must be specified.

**Reviewer #1: Yes**

**Reviewer #2: Yes**

5. Is the manuscript presented in an intelligible fashion and written in standard English?
PLOS ONE does not copyedit accepted manuscripts, so the language in submitted articles must be clear, correct, and unambiguous. Any typographical or grammatical errors should be corrected at revision, so please note any specific errors here.

**Reviewer #1: Yes**

**Reviewer #2: Yes**

**6. Review Comments to the Author**

**List of Changes – Reviewers’ Comments**

**Reviewer # 2**

**1. Reviewer:** Thank you and Appreciation to the authors for the attention given to the reviewers’ suggestions. The statistical tools used are well defended. This reviewer still has concern that the authors do not use this data to say something new or give strength to ways of dealing with the problem. The conclusion after all is not new - we all know about this phenomenon. The following comments are suggestions towards achieving this.

**Authors’ Feedback:** We thank the reviewer for the comments. In the following paragraphs, we will address all issues indicated by the Reviewer #2 and make changes to the manuscript accordingly.

**2. Reviewer:** The benefit of this paper to influence practice – while authors argue they wanted to have a clinical / clinician focus it. Assumes this will lead to change – but we have known for a long time that urgent procedures occur more often in the later part of the day with sicker people, reduced supporting services etc. Can the authors contribute more critically? If cardiology work up is so important – shouldn’t that be made more available – line 100 and 110. If this is true, why not explore this in the data.

**Authors’ Feedback:** Thanks for the suggestions. Addressing issues raised by the Reviewer in comments 2 and 3, we have rewritten the “Study Overview and Population” paragraph so its clearer and better understandable. Changes were provided in the final version of the manuscript.

Materials and Methods (page 4, line 97): “Cardiology consultation and perioperative evaluation is provided in less than 24 hours in our hospital. For this study, we excluded patients from whom it was not possible to perform perioperative cardiovascular evaluation or to obtain time of the surgery with the available documentation. We included patients undergoing all arterial vascular surgeries: open or endovascular (for aorta, peripheral artery, visceral artery, and carotid artery diseases and amputations due to limb ischemia), emergent, urgent, or elective. Of note, in the present study, urgent/emergency procedures were considered interventions for acute onset pathologies or clinical deterioration without previously schedule or outpatient evaluation that were able to receive perioperative cardiac evaluation.”

**3. Reviewer:** Along the same lines in the method it says “This is part of a prospective cohort registry including consecutive patients undergoing non-cardiac arterial vascular surgery between 2012 and 2018 at Hospital das Clínicas, University of São Paulo Medical School, São Paulo, Brazil, for whom a preoperative cardiologic consultation was requested”. What does this mean – when you have included urgent patients who didn’t get this consultation. Your addition line 99-100 makes this confusing. Perhaps you could rewrite the description of your cohort.

**Authors’ Feedback:** As stated in Item 2, changes were provided in the final version of the manuscript.

**4. Reviewer:** Is it not of interest to know who missed out - as requested in the previous peer review who were these and what the role of your preoperative assessment achieved for those who did get it? Otherwise there is little relevance of mentioning preoperative consultation to the outcome of the study.

**Authors’ Feedback:** We appreciate the commentary. We made changes that can be seen in final version of the manuscript.

Discussion (page 14, line 330): “In clinical practice, low risk patients according to anesthesiology evaluation are often directly sent to surgical theater without further consultation. Given the nature of the present study (retrospective analysis of prospective collected data) we don’t have the number of patients operated without cardiac consultation, but we estimate a small number of cases that could not influence the final results of the present study.”

**5. Reviewer:** Reference to reoperation in previous review assumed the patients and the index procedure were only counted once but rather that reoperation has other possible implications – when reoperation is the index case, and that subsequent reoperation would be an important factor in outcomes at 30 days and one year.

**Authors’ Feedback:** We agree with reviewer that reoperation could be, per se, a risk factor for complications. However, this is not the purpose of the present study, that addresses the period of the day as a potential marker of complications. Kindly answering to Reviewer #2, we looked for reoperations in our database and found only 57 patients (4.4%) that were reoperated in one year after the first surgery.

**6. Reviewer:** Line 328 Not sure what the addition (redline version) means “We may have missed patients”? see the confusion in comment 3 above. Does this mean all the patients (including the urgents) had vascular surgeon’s request made for cardiology consultation (and not all got it) and then there were a few for whom the vascular team didn’t get around to it and they are not in the study population?

**Authors’ Feedback:**  We have addressed to this issue in Itens 2 and 3. All patients included in our study had cardiology consultation and preoperative risk evaluated, including urgent/emergency surgeries. We did not include in our study patients in low risk strata, e.g., those referred to anesthesiology evaluation and directly sent to surgical theater without further consultation. We also addressed to this issue in Item 4.

**7. Reviewer:** This comes back to what is the relevance of the consultation why is cardiac consultation important. It seems to this reviewer that the authors have alluded to this in the last paragraph but unfortunately have not used the data they have to explore this e.g there presumably are at least 1000 subjects who were referred for the cardiology consultation – Another example you present data of differences in cardiac drugs - surely a benefit of seeing a cardiologist which indicates urgent cases seem not to be protected as well etc. Another example might be: Cardiac risk Index – speak of risk – can we presume this data is more likely to be documented by cardiology consultation? This reviewer suspects this is far more important than fasting time!

**Authors’ Feedback:** We thank the reviewer for the comment. The main outcome of the present study is the influence of the period of the day in the occurrence of complications and not the effect of cardiac consultation. As matter of fact, having cardiac consultation was an inclusion criteria to the study The role of cardiac consultation in this setting is also an important question that can be addressed in future studies.

**8. Reviewer:** Thank you for the additional table This is quite revealing – amputations left to the end of the day , maybe for the juniors, often very sick, AAAE mainly during the morning – good idea , LLR being done late in the day . This deserves comment.

**Authors’ Feedback:** We agree that showing this data is important for readers, but there is no power in our study to get conclusions or get clinical recommendations for each type of interventions separately, once we have not previously designed our study for those outcomes.
