# Supplementary material for: Impact of the period of the day on all-cause mortality and major cardiovascular complications after arterial vascular surgeries
Source: PLoS One. 2023 Jan 5;18(1):e0279873. doi: 10.1371/journal.pone.0279873 (PMC9815593; doi:10.1371/journal.pone.0279873)
Supplement: Attachment — Submitted filename: Response to Reviewers 09-24-2022.docx [file pone.0279873.s007.docx]

**Response to Reviewers**

**Impact of the period of the day on all-cause mortality and major cardiovascular complications after arterial vascular surgeries**

Dear Editor and Reviewers,

The first round of Review was really enriching for us. We appreciate your consideration on reviewing our manuscript. We have carefully read and analyzed your comments and provided answers and changes when necessary.

For this second round of Review, please note that changes we made in the manuscript text are marked as the “red” color. Also, we are providing the files of both marked and clean “Impact of the period of the day on all-cause mortality and major cardiovascular complications after arterial vascular surgeries” reviewed versions attached.

1. If the authors have adequately addressed your comments raised in a previous round of review and you feel that this manuscript is now acceptable for publication, you may indicate that here to bypass the “Comments to the Author” section, enter your conflict of interest statement in the “Confidential to Editor” section, and submit your "Accept" recommendation.

**Reviewer #1: All comments have been addressed**

**Reviewer #2: (No Response)**

2. Is the manuscript technically sound, and do the data support the conclusions?
The manuscript must describe a technically sound piece of scientific research with data that supports the conclusions. Experiments must have been conducted rigorously, with appropriate controls, replication, and sample sizes. The conclusions must be drawn appropriately based on the data presented.

**Reviewer #1: Yes**

**Reviewer #2: Partly**

3. Has the statistical analysis been performed appropriately and rigorously?

**Reviewer #1: Yes**

**Reviewer #2: Yes**

4. Have the authors made all data underlying the findings in their manuscript fully available?
The [PLOS Data policy](http://www.plosone.org/static/policies.action#sharing) requires authors to make all data underlying the findings described in their manuscript fully available without restriction, with rare exception (please refer to the Data Availability Statement in the manuscript PDF file). The data should be provided as part of the manuscript or its supporting information, or deposited to a public repository. For example, in addition to summary statistics, the data points behind means, medians and variance measures should be available. If there are restrictions on publicly sharing data—e.g. participant privacy or use of data from a third party—those must be specified.

**Reviewer #1: Yes**

**Reviewer #2: Yes**

5. Is the manuscript presented in an intelligible fashion and written in standard English?
PLOS ONE does not copyedit accepted manuscripts, so the language in submitted articles must be clear, correct, and unambiguous. Any typographical or grammatical errors should be corrected at revision, so please note any specific errors here.

**Reviewer #1: Yes**

**Reviewer #2: Yes**

**6. Review Comments to the Author**

**List of Changes – Reviewers’ Comments**

**Reviewer #2**: The account of who did and who didnt get cardiology consultation is at best confusing. If a patient with a rupture AAA appeared at 11 pm was cardiology consultation available or not prior to surgery? What happened to all the patients for whom the anaesthetist made decisions. Why could you not previously account for who did and who did not have consultation when you now say only those with consultation were included in the study ?

Please describe your consultation service that sees all patients within 24 hours - what hours of the day is the service not available . Why the insistence to describe this service and use it as your selection criteria and not want to explore its relevance?

**Authors’ Feedback:**

We thank the reviewer for the comment. If a patient with a rupture of AAA was admitted to the hospital and operated directly during night he would not have received a cardiac consultation and would not have been included in our cohort. In our hospital, cardiology evaluations are performed between 7AM and 6 PM.

Since our service is a referral center for complex vascular cases, a majority of patients are considered to be at intermediate or high risk of cardiac complications, therefore is common practice at our center to ask for cardiac consultation for the vast majority of procedures.

Indeed, a few low-risk and very high-risk patients (emergent surgeries) are not accounted in our cohort. Although this might be responsible for some degree of selection bias, we believe that the small number of surgeries would not impact on the general findings of our study. The high number of events that was observed in the main cohort also attenuates the effect of this lost patients.

We decided to use only patients with cardiac evaluation in our cohort to standardize data acquisition, perioperative care with the most recent guidelines and active perioperative monitoring for cardiac events for a more robust database.

Changes were made to the manuscript (lines 329-331 and 335-336) to account better address this subject.

Discussion (page 14 and 15 – Lines 329-336)

Our study has some limitations. First, our study population involves patients from a tertiary health care center to which patients are usually referred when they have a more severe vascular condition or important cardiac or noncardiac comorbidities. We included in the cohort patients for whom cardiac consultation was requested, and although it is a common practice at our institution for vascular surgeons to request cardiac evaluation for all patients, we may have missed patients that need emergent surgery and proceeded directly to the operating room without cardiac evaluation or patients that were considered to be in the lower risk strata. In clinical practice, low risk patients according to anesthesiology evaluation are often directly sent to surgical theater without further consultation. Given the nature of the present study (retrospective analysis of prospective collected data) we don’t have the number of patients operated without cardiac consultation, but we estimate a small number of cases that would be less likely to influence the final results of the present study. Also-ran, the large number of events that was observed in our cohort makes less likely the influence of a small number missed patients
