# Supplementary material for: Impact of the period of the day on all-cause mortality and major cardiovascular complications after arterial vascular surgeries
Source: PLoS One. 2023 Jan 5;18(1):e0279873. doi: 10.1371/journal.pone.0279873 (PMC9815593; doi:10.1371/journal.pone.0279873)
Supplement: Attachment — Submitted filename: Response to Reviewers 30-Nov-22.docx [file pone.0279873.s008.docx]

**Response to Reviewers**

**Impact of the period of the day on all-cause mortality and major cardiovascular complications after arterial vascular surgeries**

Dear Editor and Reviewers,

We appreciate your consideration on reviewing our manuscript. We have carefully read and analyzed your comments and provided answers and changes when necessary.

For this third round of Review, please note that changes we made in the manuscript text are marked as the “red” color. Also, we are providing the files of both marked and clean “Impact of the period of the day on all-cause mortality and major cardiovascular complications after arterial vascular surgeries” reviewed versions attached.

1. If the authors have adequately addressed your comments raised in a previous round of review and you feel that this manuscript is now acceptable for publication, you may indicate that here to bypass the “Comments to the Author” section, enter your conflict of interest statement in the “Confidential to Editor” section, and submit your "Accept" recommendation.

**Reviewer #3: (No Response)**

**Reviewer #4: All comments have been addressed**

2. Is the manuscript technically sound, and do the data support the conclusions?
The manuscript must describe a technically sound piece of scientific research with data that supports the conclusions. Experiments must have been conducted rigorously, with appropriate controls, replication, and sample sizes. The conclusions must be drawn appropriately based on the data presented.

**Reviewer #3: Partly**

**Reviewer #4: Yes**

3. Has the statistical analysis been performed appropriately and rigorously?

**Reviewer #3: Yes**

**Reviewer #4: Yes**

4. Have the authors made all data underlying the findings in their manuscript fully available?
The [PLOS Data policy](http://www.plosone.org/static/policies.action#sharing) requires authors to make all data underlying the findings described in their manuscript fully available without restriction, with rare exception (please refer to the Data Availability Statement in the manuscript PDF file). The data should be provided as part of the manuscript or its supporting information, or deposited to a public repository. For example, in addition to summary statistics, the data points behind means, medians and variance measures should be available. If there are restrictions on publicly sharing data—e.g. participant privacy or use of data from a third party—those must be specified.

**Reviewer #3: Yes**

**Reviewer #4: Yes**

5. Is the manuscript presented in an intelligible fashion and written in standard English?
PLOS ONE does not copyedit accepted manuscripts, so the language in submitted articles must be clear, correct, and unambiguous. Any typographical or grammatical errors should be corrected at revision, so please note any specific errors here.

**Reviewer #3: Yes**

**Reviewer #4: Yes**

6. Review Comments to the Author. Please use the space provided to explain your answers to the questions above. You may also include additional comments for the author, including concerns about dual publication, research ethics, or publication ethics. (Please upload your review as an attachment if it exceeds 20,000 characters)

**Reviewer #3: A very major flaw with this study is that there is no analysis made of “afternoon” surgery (ie normal working hours) and out of hours (emergent) surgery. Out of hours surgery will always have poorer outcomes given the emergent nature of the work.**

**Reviewer #4: (No Response)**

**List of Changes – Reviewers’ Comments**

**Reviewer # 3**

1. **Reviewer:** A very major flaw with this study is that there is no analysis made of “afternoon” surgery (ie normal working hours) and out of hours (emergent) surgery. Out of hours surgery will always have poorer outcomes given the emergent nature of the work.

**Authors’ Feedback:** We thank the reviewer for the comment. In the following paragraphs, we will address the collinearity test we made analyzing the endpoints in the urgent/emergency surgeries group. We made changes to the manuscript accordingly.

Materials and Methods (page 7, line 188): “Sensitivity Analysis - Since urgent/emergency surgeries are more frequent in the night period they could be correlated variables. To avoid confounding results a sensitivity analysis was made considering only the population undergoing urgent/emergency surgery, submitting them to the same statistical analysis performed in the main cohort. ”

Results (page 12, line 264): “Of the total, 478 patients underwent urgent/emergency surgery: 181 (37.9%) during the morning and 297 (62.1%) during the afternoon/night period. Considering only urgent/emergency surgery population, significant increase in all-cause mortality in the afternoon/evening surgery group was observed after 30 days (HR 2.57 [95% CI 1.69 - 3.93], P value < 0.001) and one year (HR 2 .43 [95% CI 1.75 - 3.37], P value < 0.001). After multivariate analysis, 30-day all-cause mortality was higher in the afternoon/night period (aHR 2.43 [95%CI 1.54 - 3.83], P = < 0.001), as well as one year mortality (aHR 2.35 [95%CI 1.65 – 3.33], P =< 0.001 – S3 Fig. Mortality in Urgent/Emergency Surgeries).

The incidence of PMI in the urgent/emergency surgery group was also higher in patients operated in the afternoon/evening period: 44.2% versus 33.0%, respectively (OR 1.82 [95%CI 1.22 - 2.71], P- value = 0.003). In the multivariate analysis, urgent/emergency surgeries performed in the afternoon/evening presented independent association with the incidence of PMI (aHR 1.4 [95%CI 1.03 – 1.91], P =0.03). After 30 days, the incidence of MACE in urgent/emergency surgery group was higher in the afternoon/evening surgery population. Surgeries performed in the afternoon/evening period were independently associated with increased MACE after correction for confounding factors in the multivariable analysis (aHR 1.51 [95%CI 1.10 – 2.06], P =0.010).”

Supporting Material (page 21, line 526): S3 Fig. Mortality in Urgent/Emergency Surgeries

**S3 Fig. Mortality in Urgent/Emergency Surgeries**
